# Exploration of multiphoton entangled states by using weak nonlinearities

**DOI:** 10.1038/srep19116

**Published:** 2016-01-11

**Authors:** Ying-Qiu He, Dong Ding, Feng-Li Yan, Ting Gao

**Affiliations:** 1 College of Physics Science and Information Engineering, Hebei Normal University, Shijiazhuang 050024, China; 2 Department of Basic Curriculum, North China Institute of Science and Technology, Beijing 101601, China; 3 College of Mathematics and Information Science, Hebei Normal University, Shijiazhuang 050024, China

## Abstract

We propose a fruitful scheme for exploring multiphoton entangled states based on linear optics and weak nonlinearities. Compared with the previous schemes the present method is more feasible because there are only small phase shifts instead of a series of related functions of photon numbers in the process of interaction with Kerr nonlinearities. In the absence of decoherence we analyze the error probabilities induced by homodyne measurement and show that the maximal error probability can be made small enough even when the number of photons is large. This implies that the present scheme is quite tractable and it is possible to produce entangled states involving a large number of photons.

Undoubtedly, entanglement^1^,^2^,^3^,^4^ is one of the most crucial elements in quantum information processing, e.g. quantum cryptography^5^, quantum teleportation^6^, quantum secure direct communication^7^,^8^, etc. In recent years, quantum entanglement has been extensively investigated in various candidate physical systems^9^,^10^,^11^,^12^,^13^,^14^,^15^,^16^, in particular, one can prepare and manipulate multipartite entanglement in optical systems^17^,^18^,^19^,^20^,^21^,^22^,^23^,^24^,^25^,^26^,^27^,^28^.

Generally, a spontaneous parametric down-conversion (PDC) source^29^,^30^ is capable of emitting pairs of strongly time-correlated photons in two spatial modes. As extensions of interest, with linear optics and nonlinear optical materials several schemes for creating multiphoton entangled states have been proposed^31^,^32^,^33^,^34^,^35^,^36^,^37^. For a large number of photons, however, there are some technological challenges such as probabilistic emission of PDC sources and imperfect detectors. A feasible approach is to use the simple single-photon sources, instead of waiting the successive pairs, and quantum nondemolition (QND) measurement^38^,^39^,^40^,^41^,^42^,^43^,^44^,^45^ with weak Kerr nonlinearities. Note that the Kerr nonlinearities^46^,^47^,^48^ are extremely weak and the order of magnitude of them is only 10^−2^ even by using electromagnetically induced transparency^49^,^50^. More recently, Shapiro *et al.*^51^,^52^ showed that the causality-induced phase noise will preclude high-fidelity *π*-radian conditional phase shifts created by the cross-Kerr effect. In these cases, with the increase of the number of photons it is usually more and more difficult to study multiphoton entanglement in the regime of weak nonlinearities.

In this paper, we focus on the exploration of multiphoton entangled states with linear optics and weak nonlinearities. We show a quantum circuit to evolve multimode signal photons fed by a group of arbitrary single-photon states and the coherent probe beam. Particularly, there are only two specified but small phase shifts induced in the process of interaction with weak nonlinearities. This fruitful architecture allows us to explore multiphoton entangled states with a large number of photons but still in the regime of weak nonlinearities.

## Kerr nonlinearities

Before describing the proposed scheme, let us first give a brief introduction of the Kerr nonlinearities. The nonlinear Kerr media can be used to induce a cross phase modulation with Hamiltonian of the form 
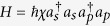
, where 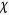 is the coupling constant and 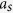 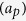 represents the annihilation operator for photons in the signal (probe) mode. If we assume that the signal mode is initially described by the state 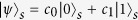 and the coherent probe beam is 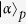, then after the Kerr interaction the whole system evolves as


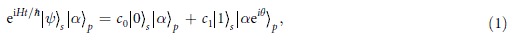


where 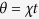 with interaction time *t*. In order to distinguish different cases, one may perform a homodyne measurement^39^ on the probe beam with quadrature operator 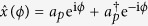, where 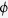 is a real constant. Especially for 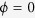, this operation is conventionally referred to as *X* homodyne measurement; while for 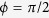, it is called *P* homodyne measurement.

## Creation of multiphoton entangled states with linear optics and weak nonlinearities

**Figure 1:**
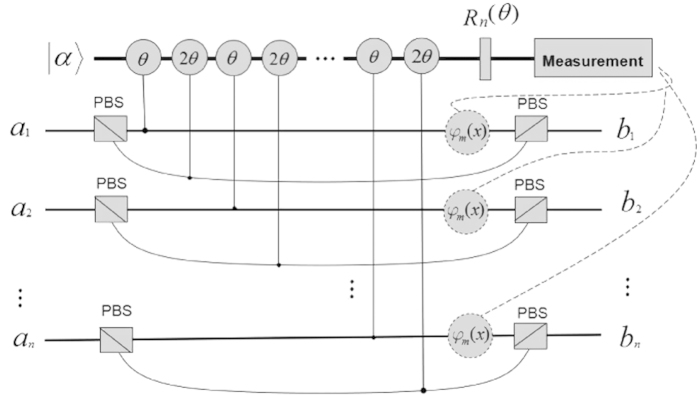
The schematic diagram of creating multiphoton entangled states with linear optics and weak nonlinearities. 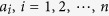
 are input ports and each port is supplied with an arbitrary single-photon state, while 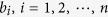 are the corresponding outputs, respectively. *θ* and 2*θ* represent phase shifts in the coherent probe beam 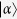 induced by Kerr interaction between photons. 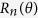 is a phase shift gate. Each 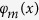 represents a phase shift on the signal photons based on classical feed-forward information.

Let 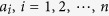 represent input ports with respective spatial modes, namely signal modes, and 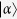 is a coherent beam in probe mode. The setup of creating multiphoton entangled states is shown in Fig. 1.

Without loss of generality we may suppose that each input port is supplied with an arbitrary single-photon state. Then, the total input state reads


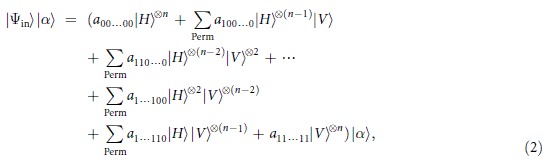


where 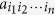, 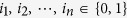, are complex coefficients satisfying the normalization condition 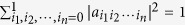 and 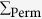 denotes the sum over all possible permutations of the signal modes, for example,


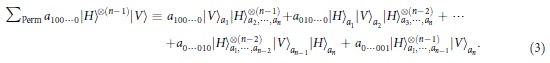


Each polarizing beam splitter (PBS) is used to transmit 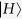 polarization photons and reflect 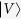 polarization photons. When the signal photons travel to the PBSs, they will be individually split into two spatial modes and then interact with the nonlinear media so that pairs of phase shifts *θ* and 2*θ* are induced on the coherent probe beam, respectively. We here introduce a single phase gate 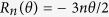 so as to implement the next *X* homodyne measurement on the probe beam. 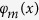, 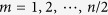 for even *n* and 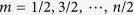 for odd *n*, are phase shifts on the signal photons based on the measured values of *x* via the classical feed-forward information. At last, at the ports 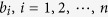 one may obtain 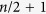 output states for even *n* or 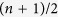 states for odd *n*.

We describe our method in details. For *n* is even, after the interaction between the photons with Kerr media and followed by the action of the phase gate, the combined system evolves as


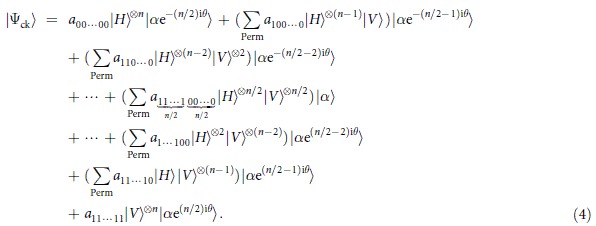


In order to create the desired multiphoton entangled states, we here perform an *X* homodyne measurement^38^,^45^ on the probe beam. If the value *x* of the *X* homodyne measurement is obtained, then the signal photons become


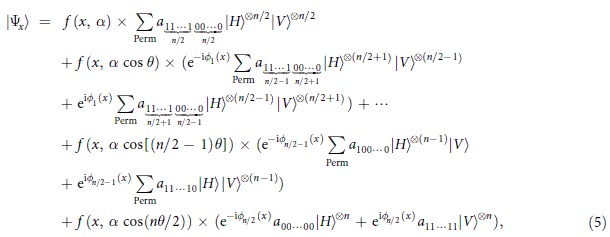


where


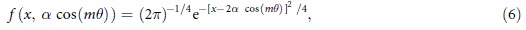



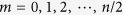
, are respectively Gaussian curves which are associated with the probability amplitudes of the outputs, and


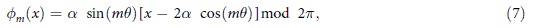



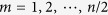
, are respectively phase factors based on the values of the *X* homodyne measurement. Note that the peaks of these Gaussian distributions locate at 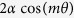. Thus, the midpoints between two neighboring peaks 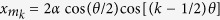 and the distances of two nearby peaks 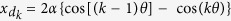 with 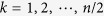. Obviously, with these 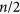 midpoint values 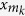 there exist 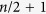 intervals and each interval corresponds to an output state.

We now consider the phase shifts 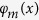. The signal photon evolves as 
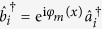
, 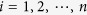. A straightforward calculation shows that


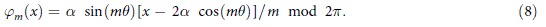


After these feed-forward phase shifts have been implemented and the signal photons pass through the PBSs, one can obtain the desired states as follows. Clearly, for 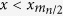 we have


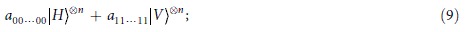


for 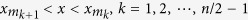, we obtain the states


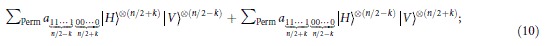


and for 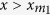 we obtain the state


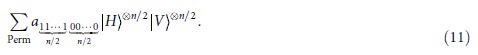


Similarly, for odd *n*, we have


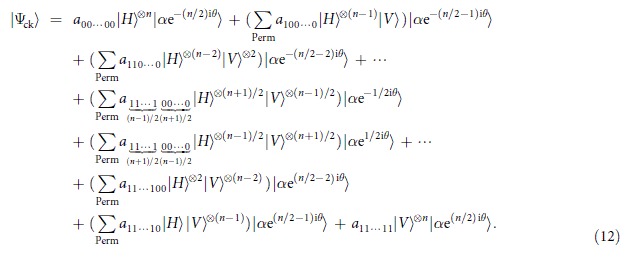


Also,


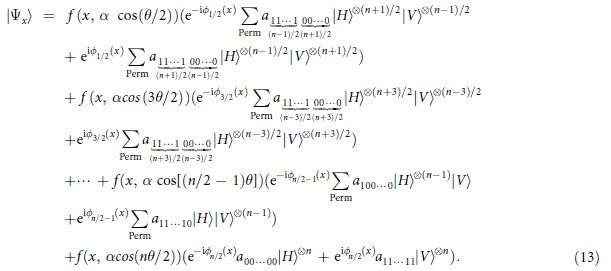


Here, the functions 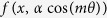, phase shifts 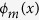 and 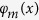 are approximately the same as those described for even *n*, except for 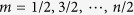, and then the similar results hold for the midpoints 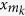 and the distances 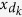 with 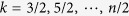. Of course, with 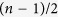 midpoint values 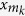 there may be 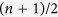 output states; that is, one can obtain the states 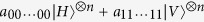 for


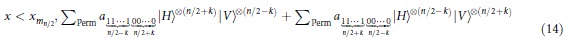


for


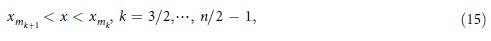


and


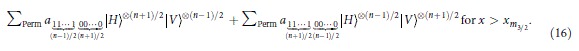


As an example of the applications of interest for the present scheme, we introduce a class of remarkable multipartite entangled states


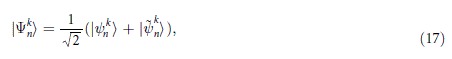


where


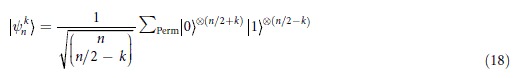


and


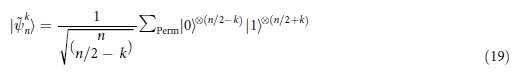


are two orthonormal states, namely Dicke states^53^,^54^. In view of its “catness”, the state 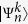 can be referred to as *cat-like state*, and especially for 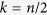 it can be expressed as the canonical *n*-partite Greenberger-Horne-Zeilinger (GHZ) state. In the present scheme, obviously, for 
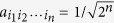
, 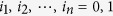, we can obtain these cat-like states with 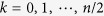 for even *n* and 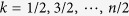 for odd *n*, where the qubits are encoded with the polarization modes 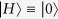 and 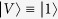. Of course, more generally, we may project out a group of multiphoton entangled states involving generalized Dicke states.

## Discussion

There are two models commonly employed in the process of Kerr interaction, single-mode model and continuous-time multi-mode model^51^. The former implies that one may ignore the temporal behavior of the optical pulses but the latter is causal, non-instantaneous model involving phase noise. It has been shown that^52^ this causality-induced phase noise will preclude the possibility of high-fidelity CPHASE gates created by the cross-Kerr effect in optical fiber. To solve this problem, one may need to find an optimum response function for the available medium, or to exploit more favorable systems, such as cavitylike systems^55^. After all, the ultimate possible performance of Kerr interaction with a larger system is an interesting open issue. More recently, we note that Feizpour *et al.*^56^ showed the first direct measurement of the cross-phase shift due to single photons. It may be possible to open a door for future studies of nonlinear optics in quantum information processing. In the present scheme, we restrict ourselves to ignoring the phase noise and concentrate mainly on showing a method for exploring multiphoton entangled states in the regime of weak cross-Kerr nonlinearities, i.e. 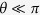.

It is worth noting that, there are only small phase shifts *θ* and 2*θ* instead of a series of related functions of the number of photons in the process of interaction with Kerr nonlinearities. This implies that the present scheme is quite tractable especially for creating entangled states with a larger number of photons. In addition, the error probabilities 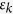 are 
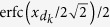
, which come from small overlaps between two neighboring curves. Considering the distances of two nearby peaks 
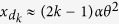
 with 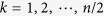 for even *n* and 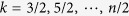 for odd *n*, the maximal error probability 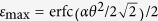, which is exactly the result described by Nemoto and Munro in^40^. Obviously, the error probabilities in our scheme are no more than that one even when the number of photons is large. Therefore, by choosing an appropriate coherent probe beam the error probability can be reduced to as low a level as desired and then the present scheme may be realized in a nearly deterministic manner.

In summary, based on linear optics and weak nonlinearities we have shown a fruitful method for exploring a class of multiphoton entangled states, the *generalized cat-like states*. Evidently, three aspects are noteworthy in the present framework. First, since there are no large phase shifts in the interacting process with weak Kerr nonlinearities, our scheme is more feasible compared with the previous schemes. Second, the system is measured only once with a small error probability and it means that the present scheme might be realized near deterministically. Finally, the fruitful architecture allows us to explore a group of multiphoton entangled states involving a large number of photons, i.e., to produce entangled states approaching the macroscopic domain.

## Additional Information

**How to cite this article**: He, Y.-Q. *et al.* Exploration of multiphoton entangled states by using weak nonlinearities. *Sci. Rep.* **6**, 19116; doi: 10.1038/srep19116 (2016).

## References

[b1] Horodecki R., Horodecki P., Horodecki M. & Horodecki K. Quantum entanglement. Rev. Mod. Phys. 81, 865–942 (2009).

[b2] Gühne O. & Tóth G. Entanglement detection. Phys. Rep. 474, 1–75 (2009).

[b3] Yan F. L., Gao T. & Chitambar E. Two local observables are sufficient to characterize maximally entangled states of *N* qubits. Phys. Rev. A 83, 022319 (2011).

[b4] Gao T., Yan F. L. & van Enk S. J. Permutationally invariant part of a density matrix and nonseparability of *N*-qubit states. Phys. Rev. Lett. 112, 180501 (2014). 24856681 10.1103/PhysRevLett.112.180501

[b5] Ekert A. K. Quantum cryptography based on Bell’s theorem. Phys. Rev. Lett. 67, 661–663 (1991). 10044956 10.1103/PhysRevLett.67.661

[b6] Bennett C. H. *et al.* Teleporting an unknown quantum state via dual classical and Einstein-Podolsky-Rosen channels. Phys. Rev. Lett. 70, 1895–1899 (1993). 10053414 10.1103/PhysRevLett.70.1895

[b7] Long G. L. & Liu X. S. Theoretically efficient high-capacity quantum-key-distribution scheme. Phys. Rev. A 65, 032302 (2002).

[b8] Deng F. G., Long G. L. & Liu X. S. Two-step quantum direct communication protocol using the Einstein-Podolsky-Rosen pair block. Phys. Rev. A 68, 042317 (2003).

[b9] Nielsen M. A. & Chuang I. L. Quantum Computation and Quantum Information (Cambridge University Press, Cambridge, 2000).

[b10] Waks E. & Monroe C. Protocol for hybrid entanglement between a trapped atom and a quantum dot. Phys. Rev. A 80, 062330 (2009).

[b11] Gonzalez-Tudela A. *et al.* Entanglement of two qubits mediated by one-dimensional plasmonic waveguides. Phys. Rev. Lett. 106, 020501 (2011). 21405211 10.1103/PhysRevLett.106.020501

[b12] Shwa D., Cohen R. D., Retzker A. & Katz N. Heralded generation of Bell states using atomic ensembles. Phys. Rev. A 88, 063844 (2013).

[b13] Casabone B. *et al.* Heralded entanglement of two ions in an optical cavity. Phys. Rev. Lett. 111, 100505 (2013). 25166644 10.1103/PhysRevLett.111.100505

[b14] Feng G. R., Long G. L. & Laflamme R. Experimental simulation of anyonic fractional statistics with an NMR quantum-information processor. Phys. Rev. A 88, 022305 (2013).

[b15] Sheng Y. B., Deng F. G. & Long G. L. Complete hyperentangled-Bell-state analysis for quantum communication. Phys. Rev. A 82, 032318 (2010).

[b16] Liu S. P., Li J. H., Yu R. & Wu Y. Achieving three-dimensional entanglement between two spatially separated atoms by using the quantum Zeno effect. Phys. Rev. A 87, 062316 (2013).

[b17] Scully M. O. & Zubairy M. S. Quantum Optics (Cambridge University Press, Cambridge, 1997).

[b18] Knill E., Laflamme R. & Milburn G. J. A scheme for efficient quantum computation with linear optics. Nature 409, 46–52 (2001). 11343107 10.1038/35051009

[b19] Kok P. *et al.* Linear optical quantum computing with photonic qubits. Rev. Mod. Phys. 79, 135–174 (2007).

[b20] Pan J. W. *et al.* Multiphoton entanglement and interferometry. Rev. Mod. Phys. 84, 777–838 (2012).

[b21] Li J. H. *et al.* Optical-frequency-comb generation and entanglement with low-power optical input in a photonic molecule. Phys. Rev. A 90, 033830 (2014).

[b22] Lü X. Y. *et al.* Quantum-criticality-induced strong Kerr nonlinearities in optomechanical systems. Sci. Rep. 3, 2943 (2013). 24126279 10.1038/srep02943 PMC3796743

[b23] Ren B. C., Wang G. Y. & Deng F. G. Universal hyperparallel hybrid photonic quantum gates with dipole-induced transparency in the weak-coupling regime. Phys. Rev. A 91, 032328 (2015).

[b24] Sheng Y. B. & Deng F. G. Deterministic entanglement purification and complete nonlocal Bell-state analysis with hyperentanglement. Phys. Rev. A 81, 032307 (2010).

[b25] Sheng Y. B., Zhou L., Zhao S. M. & Zheng B. Y. Efficient single-photon-assisted entanglement concentration for partially entangled photon pairs. Phys. Rev. A 85, 012307 (2012).

[b26] Deng F. G. Optimal nonlocal multipartite entanglement concentration based on projection measurements. Phys. Rev. A 85, 022311 (2012).

[b27] Sheng Y. B. & Zhou L. Deterministic entanglement distillation for secure double-server blind quantum computation. Sci. Rep. 5, 7815 (2015). 25588565 10.1038/srep07815 PMC4295105

[b28] Sheng Y. B. & Zhou L. Two-step complete polarization logic Bell-state analysis. Sci. Rep. 5, 13453 (2015). 26307327 10.1038/srep13453 PMC4549687

[b29] Kwiat P. G. *et al.* New high-intensity source of polarization-entangled photon pairs. Phys. Rev. Lett. 75, 4337–4341 (1995). 10059884 10.1103/PhysRevLett.75.4337

[b30] Simon C. & Bouwmeester D. Theory of an entanglement laser. Phys. Rev. Lett. 91, 053601 (2003). 12906596 10.1103/PhysRevLett.91.053601

[b31] Bouwmeester D. *et al.* Observation of three-photon Greenberger-Horne-Zeilinger entanglement. Phys. Rev. Lett. 82, 1345–1349 (1999).

[b32] Pan J. W. *et al.* Experimental demonstration of four-photon entanglement and high-fidelity teleportation. Phys. Rev. Lett. 86, 4435–4438 (2001). 11384253 10.1103/PhysRevLett.86.4435

[b33] Eisenberg H. S. *et al.* Quantum entanglement of a large number of photon. Phys. Rev. Lett. 93, 193901 (2004). 15600835 10.1103/PhysRevLett.93.193901

[b34] Walther P., Aspelmeyer M. & Zeilinger A. Heralded generation of multiphoton entanglement. Phys. Rev. A 75, 012313 (2007).

[b35] Wei T. C., Lavoie J. & Kaltenbaek R. Creating multiphoton-polarization bound entangled states. Phys. Rev. A 83, 033839 (2011).

[b36] Tichy M. C., Mintert F. & Buchleitner A. Limits to multipartite entanglement generation with bosons and fermions. Phys. Rev. A 87, 022319 (2013).

[b37] He Y. Q., Ding D., Yan F. L. & Gao T. Preparation and purification of four-photon Greenberger-Horne-Zeilinger state. J. Phys. B: At. Mol. Opt. Phys. 48, 055501 (2015).

[b38] Barrett S. D. *et al.* Symmetry analyzer for nondestructive Bell-state detection using weak nonlinearities. Phys. Rev. A 71, 060302 (2005).

[b39] Munro W. J., Nemoto K., Beausoleil R. G. & Spiller T. P. High-efficiency quantum-nondemolition single-photon-number-resolving detector. Phys. Rev. A 71, 033819 (2005).

[b40] Nemoto K. & Munro W. J. Nearly deterministic linear optical controlled-NOT gate. Phys. Rev. Lett. 93, 250502 (2004). 15697884 10.1103/PhysRevLett.93.250502

[b41] Munro W. J., Nemoto K. & Spiller T. P. Weak nonlinearities: a new route to optical quantum computation. New J. Phys. 7, 137 (2005).

[b42] Kok P. Effects of self-phase-modulation on weak nonlinear optical quantum gates. Phys. Rev. A 77, 013808 (2008).

[b43] Ding D., Yan F. L. & Gao T. Preparation of *km*-photon concatenated Greenberger-Horne-Zeilinger states for observing distinctive quantum effects at macroscopic scales. J. Opt. Soc. Am. B 30, 3075–3078 (2013).

[b44] Ding D., Yan F. L. & Gao T. Entangler and analyzer for multiphoton Greenberger-Horne-Zeilinger states using weak nonlinearities. Sci. China-Phys. Mech. Astron. 57, 2098–2103 (2014).

[b45] He Y. Q., Ding D., Yan F. L. & Gao T. Exploration of photon-number entangled states using weak nonlinearities. Opt. Express 23, 21671–21677 (2015). 26368146 10.1364/OE.23.021671

[b46] Imoto N., Haus H. A. & Yamamoto Y. P. Quantum nondemolition measurement of the photon number via the optical Kerr effect. Phys. Rev. A 32, 2287–2292 (1985). 9896342 10.1103/physreva.32.2287

[b47] Rokhsari H. & Vahala K. J. Observation of Kerr nonlinearity in microcavities at room temperature. Opt. Lett. 30, 427–429 (2005). 15762450 10.1364/ol.30.000427

[b48] Matsuda N. *et al.* Observation of optical-fibre Kerr nonlinearity at the single-photon level. Nature Photon. 3, 95–98 (2009).

[b49] Schmidt H. & Imamoğlu A. Giant Kerr nonlinearities obtained by electromagnetically induced transparency. Opt. Lett. 21, 1936–1938 (1996). 19881851 10.1364/ol.21.001936

[b50] Lukin D. & Imamoğlu A. Controlling photons using electromagnetically induced transparency. Nature 413, 273–276 (2001). 11565022 10.1038/35095000

[b51] Shapiro J. H. & Razavi M. Continuous-time cross-phase modulation and quantum computation. New J. Phys. 9, 16 (2007).

[b52] Dove J., Chudzicki C. & Shapiro J. H. Phase-noise limitations on single-photon cross-phase modulation with differing group velocities. Phys. Rev. A 90, 062314 (2014).

[b53] Dicke R. H. Coherence in spontaneous radiation processes. Phys. Rev. 93, 99–110 (1954).

[b54] Thiel C. *et al.* Generation of symmetric Dicke states of remote qubits with linear optics. Phys. Rev. Lett. 99, 193602 (2007). 18233076 10.1103/PhysRevLett.99.193602

[b55] Chudzicki C., Chuang I. L. & Shapiro J. H. Deterministic and cascadable conditional phase gate for photonic qubits. Phys. Rev. A 87, 042325 (2013).

[b56] Feizpour A., Hallaji M., Dmochowski G. & Steinberg A. M. Observation of the nonlinear phase shift due to single post-selected photons. Nature Phys. 11, 905–909 (2015).

